# Intergenerational chronic undernutrition pattern and determinants in Ethiopia: a multilevel and spatial analysis of EDHS data (2000–2016)

**DOI:** 10.3389/fnut.2025.1537348

**Published:** 2025-06-19

**Authors:** Mekuriaw Nibret Aweke, Asebe Hagos, Moges Tadesse Abebe, Amare Kassaw, Molla Azmeraw, Nigusu Worku, Tesfahun Zemene Tafere, Mekides Engeda Alemu, Amanuel Tesera Alemu, Demewoz Kefale

**Affiliations:** ^1^Department of Human Nutrition, Institute of Public Health, College of Medicine and Health Sciences, University of Gondar, Gondar, Ethiopia; ^2^Department of Health Systems and Policy, Institute of Public Health, College of Medicine and Health Science, University of Gondar, Gondar, Ethiopia; ^3^Department of Pediatric and Child Health Nursing, College of Health Science, Debark University, Debark, Ethiopia; ^4^Department of Pediatrics and Child Health Nursing, College of Medicine and Health Sciences, Debre Tabor University, Debre Tabor, Ethiopia; ^5^Department of Nursing, College of Health Science, Woldia University, Woldiya, Ethiopia; ^6^Department of Surgical Nursing, School of Nursing and Midwifery, College of Medicine and Health Sciences, Debrebrhan University, Debre Birhan, Ethiopia; ^7^University of Gondar Comprehensive Specialized Hospital, Gondar, Ethiopia

**Keywords:** intergenerational chronic undernutrition, spatial analysis, multilevel analysis, Ethiopian, EDHS, malnutrition determinants

## Abstract

**Introduction:**

Intergenerational chronic undernutrition is a condition where both mothers and children experience poor nutrition, leading to a cycle of malnutrition that affects multiple generations. In Ethiopia, chronic undernutrition is a major public health challenge, impacting the health of both mothers and children and contributing to high rates of child mortality and stunting. This study aims to explore various individual, community, and environmental factors that contribute to intergenerational chronic malnutrition, focusing on regional differences and identifying targeted interventions to break the cycle of malnutrition.

**Methods:**

This study uses a cross-sectional design based on data from the Ethiopian Demographic and Health Surveys, conducted in 2000, 2005, 2011, and 2016, with a total of 30,667 participants. Spatial analysis and a multilevel binary logistic regression model were employed to identify geographical variations and potential risk factors for Intergenerational chronic undernutrition among mothers with children aged 0–59 months in Ethiopia. We used ArcMap to assess spatial patterns of undernutrition through Global Moran’s I, identify hotspots with Getis-Ord Gi*, and apply interpolation to estimate values in unsampled areas. Variables with *p*-values less than 0.2 in the bivariable analysis were included in the multivariable model. Statistical significance was determined at a 5% level. All analyses were performed using STATA 17. We used the intraclass correlation coefficient (ICC) to assess cluster variance, the median odds ratio (MOR) to evaluate heterogeneity, and the proportional change in variance (PCV) to monitor variance changes across models.

**Result:**

From 2000 to 2016, a total of weighted 33,445 samples of mothers with children aged 0–59 months and their households were included in the analysis. The overall prevalence of intergenerational chronic undernutrition was 19.09% (95% CI: 18.68–19.52%). Hotspot areas for intergenerational chronic undernutrition were primarily in northern and northeastern Ethiopia, including Tigray, Amhara, and parts of Afar. Spatial scan analysis revealed a major cluster covering most of Amhara, Tigray, Afar, and northern Somali. Several factors were significantly associated with intragenerational chronic malnutrition. Female children (AOR = 0.93; 95% CI: 0.86–0.99), maternal education at secondary level or higher (AOR = 0.42; 95% CI: 0.32–0.54), and higher household wealth status (AOR = 0.86; 95% CI: 0.76–0.96) were negatively associated with intergenerational chronic undernutrition. In contrast older child age (AOR = 3.93; 95% CI: 3.37–4.58), use of unimproved toilet facilities (AOR = 1.26; 95% CI: 1.08–1.46) and residence in the Amhara region were positively associated with intergenerational chronic undernutrition. Moreover, children from Somali, Oromia, Harari, Gambela, Addis Ababa, and Dire Dawa regions were less likely to experience intergenerational chronic undernutrition compared to those from Tigray.

**Conclusion:**

This analysis identifies factors influencing intergenerational chronic undernutrition in Ethiopia, with higher prevalence in the north and northeast Ethiopia. Female children, higher maternal education level, and higher wealth status reduce risk of intergenerational chronic undernutrition, while older age, unimproved sanitation, and living in Amhara increase the risk. Participants residing in Somali, Oromia, Harari, Gambela, Addis Ababa, and Dire Dawa had lower risk compared to children residing in Tigray. Improving education, enhancing sanitation facilities, and addressing regional inequalities are crucial steps in tackling Intergenerational chronic undernutrition in Ethiopia.

## 1 Introduction

Intergenerational malnutrition refers to the transmission of nutritional deficiencies and their consequences across generations, impacting both physical and mental health outcomes (1). Maternal nutrition significantly influences the health and development of offspring, with effects that can persist for multiple generations (2). The cycle of malnutrition frequently begins during pregnancy. Infants born to malnourished mothers are often undernourished at birth or quickly become malnourished, significantly increasing their risk of early morbidity and mortality (3). Early onset of malnutrition can lead to stunted growth later in life (4). Shorter maternal height is often associated with a higher risk of giving birth to a child who is also stunted (5).

Intergenerational chronic undernutrition (ICU) is a condition characterized by short stature in both mother and child, reflecting a cycle of poor health and nutritional status passed across generations. Short stature, a common manifestation of ICU, continues this cycle, with each generation’s nutritional deficits affecting the next generation (3, 6). Beyond physical growth, this cycle of malnutrition also impairs cognitive development, which in turn affects educational outcomes and future socioeconomic opportunities (7). Well-nourished women and girls are healthier, more empowered, and better able to contribute to society, breaking the intergenerational cycle of malnutrition (8).

Intergenerational chronic undernutrition is driven by a complex interplay of maternal, socioeconomic, and environmental factors (5, 9). These include shared genetic traits, epigenetic modifications, metabolic programming, and physical constraints on fetal growth due to reduced uterine space (9, 10). Malnourished mothers, particularly those with insufficient micronutrient intake or poor health are more likely to give birth of stunted children (10). Additionally, socio-cultural factors play a significant role, such as the transmission of poverty across generations and cultural practices like “eating down” during pregnancy, driven by the fear of giving birth to a large baby (5). Poor infant and young child feeding practices, combined with food insecurity, poverty, limited access to healthcare, and unhealthy environment contribute to malnutrition, which is further influenced by socioeconomic, commercial, and political factors and aggravates the intergenerational cycle of malnutrition (11, 12). Additionally, poor sanitation and limited access to clean water increase the risk of infections that hinder nutrient absorption, worsening the nutritional status of women and children (13, 14).

In low- and middle-income countries, maternal and child malnutrition is a complex issue that not only includes widespread undernutrition but also an increasing challenge with overweight and obesity (11). Chronic undernutrition remains a critical public health issue in Ethiopia, with significant impacts on both mothers and children. High rates of chronic undernutrition among children under five, along with maternal malnutrition, underscore ongoing challenges such as food insecurity, inadequate access to healthcare, and insufficient maternal nutrition (15). Undernutrition accounts for 45 percent of child mortality among children under the age of five in Ethiopia (16). Over 5.4 million children in this age group are affected by stunting, with approximately two million (one in ten) children experiencing growth impairment. This alarming statistic highlights the severe impact of malnutrition on child health, particularly in low socioeconomic communities.

The prevalence of stunting among children decreased from 47% in 2005 to 39% in 2016, while undernutrition among mothers reduced from 30.5% in the 2000 EDHS to 26.9% in the 2005 survey (17, 18). Although there has been progress in reducing undernutrition among children and women in Ethiopia, the burden of ICU remains complex and extensive (19). It continues to affect health, hinder cognitive development, and limit economic productivity, creating long-term challenges for individuals and the country as a whole. The economic impact of malnutrition is significant, influencing both individual well-being and overall societal progress. At a national level, the productivity losses and increased healthcare expenses associated with malnutrition can lead to a reduction in a country’s GDP by up to 16%, equating to a loss of approximately US$4.7 billion (20).

Ethiopia has implemented key initiatives to reduce intergenerational undernutrition including the Seqota Declaration (2016), National Nutrition Program (NNP), Sustainable Undernutrition Reduction in Ethiopia (SURE), Productive Safety Net Programme (PSNP) and the Food Security Strategy (21–23). These programs focus on multisectoral action, integrating nutrition and agriculture, and addressing food security.

Although Ethiopia has made progress in reducing child stunting and maternal undernutrition, there remains limited evidence on how community and environmental factors contribute to the spatial variation of ICU. This gap restricts the ability to design geographically targeted interventions that effectively address local needs. Most existing studies have focused primarily on individual-level determinants of malnutrition. This lack of comprehensive analysis limits understanding of how broader contextual factors interact to increase the burdens malnutrition across different regions in Ethiopia. By examining the multifactorial drivers of malnutrition across different regions, this study aims to uncover the complex interplay of individual, community, and environmental factors contributing to ICU. Through a multilevel and spatial analysis of EDHS data from 2000 to 2016, we seek to identify key determinants of ICU, explore regional variations, and inform targeted interventions that can break the cycle of malnutrition and improve health outcomes for future generations. This study is vital not only for understanding the underlying causes of chronic malnutrition in Ethiopia but also for guiding policy and public health strategies. By addressing the root causes of ICU, it is possible to reduce the long-term consequences of malnutrition, enhance the quality of life for affected populations, and foster sustainable economic development.

## 2 Methods

### 2.1 Study design and data source

**Figure 1:**
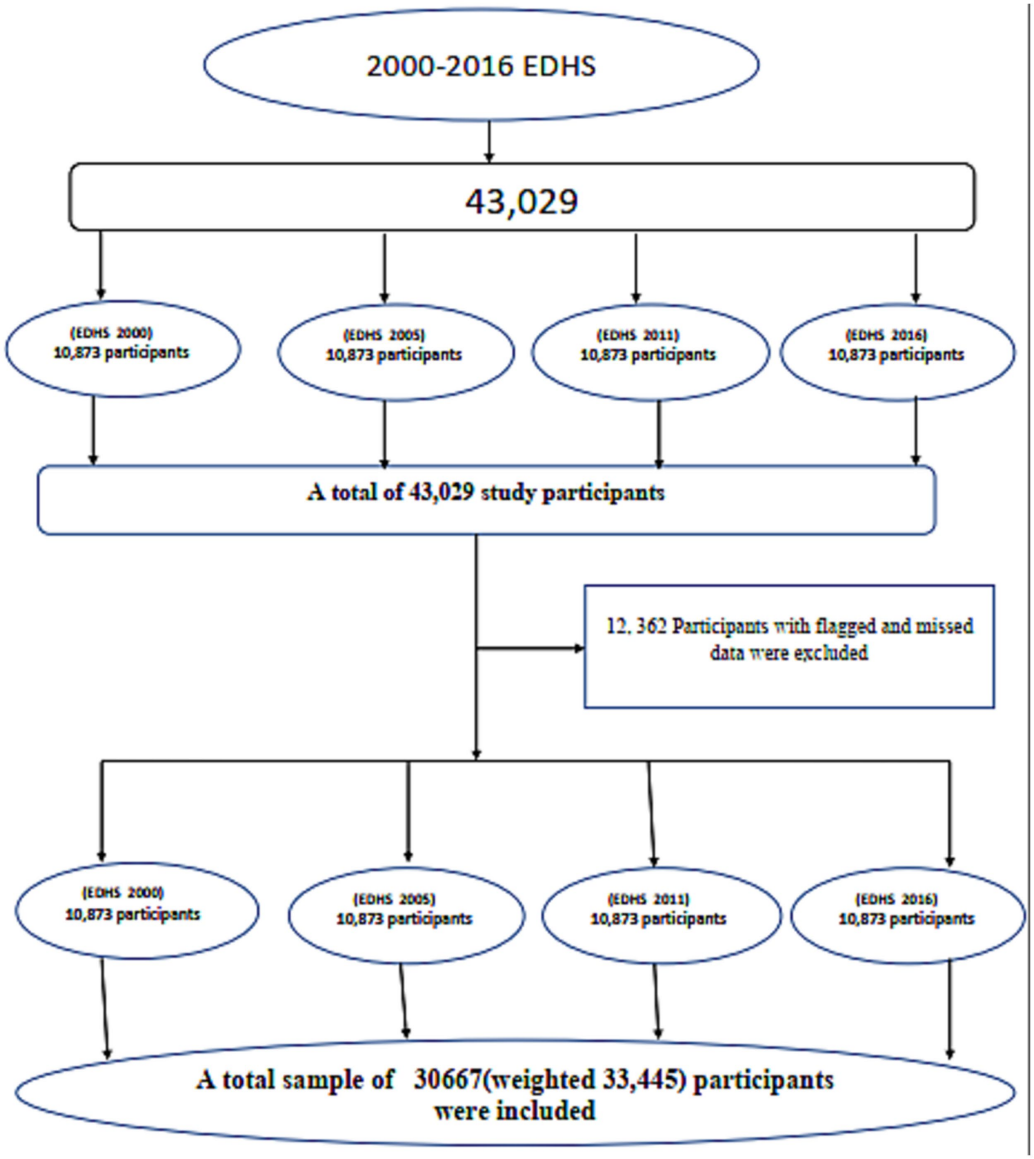
A schematic representation of the sampling procedure for the study on ICU among mothers with children aged 0–59 months in Ethiopia, 2000–2016.

This study uses a cross-sectional design based on data from the Ethiopian Demographic and Health Surveys (EDHS), conducted in 2000 with 8,574 participants, 2005 with 3,854 participants, 2011 with 9,573 participants, and 2016 with 8,666 participants, as illustrated in Figure 1. The EDHS are nationally representative studies that gather extensive data on health and demographic indicators, such as maternal and child nutrition, healthcare access, and socioeconomic factors. Ethiopia’s Central Statistical Agency (CSA) in collaboration with the Ministry of Health and USAID, the surveys provide publicly accessible and reliable data for national health and demographic assessments (24). The EDHS employs a two-stage stratified cluster sampling method. In the first stage, enumeration areas (EAs) are selected independently from each stratum, with proportional allocation based on region and residence type (urban or rural). In the second stage, a fixed number of households is systematically sampled from the selected EAs in each survey year. Ethiopia Demographic and Health Survey geographic displacement procedures were applied to safeguard the confidentiality of participants. The GPS coordinates of survey clusters were randomly displaced by up to 2 kilometers in urban areas and up to 5 kilometers in rural areas.

### 2.2 Study setting and study population

The study took place in Ethiopia, a country in the Horn of Africa, located between 3° and 14.8°N latitude and 33° and 48°E longitude. Ethiopia shares borders with Somalia, Sudan, Djibouti, Kenya, and Eritrea, spanning a total border length of 5,311 km. It is the 10th largest country in Africa and the second most populous, with over 115 million people. The country is divided into nine regions Tigray, Afar, Amhara, Oromia, Somalia, Benishangul-Gumuz, SNNPR (Southern Nations, Nationalities, and Peoples Region), Gambela, and Harari as well as two self-governed cities, Addis Ababa and Dire Dawa (25, 26). This study focused on women aged 15 to 49 years and their children under five to explore ICU in Ethiopia. It analyzed mother-child pairs, using data on mothers’ height and children’s growth measurements like height-for-age. Mothers with children with missing or unrealistic data were excluded, leaving a final sample of over 30,667 pairs (weighted to 33,445) from the four survey rounds (27–29).

### 2.3 Study variables

#### 2.3.1 Dependent variable

In this study, the dependent variable is ICU, a binary classification variable used to assess ICU within households. Intergenerational chronic undernutrition is assigned a value of 1 when both the mother and child are stunted. Maternal stunting is defined as a height of less than 155 cm (30, 31), while child stunting is determined by a height-for-age z-score (HFA) below −2. Conversely, ICU is assigned a value of 0 when either the mother, the child, or both are not stunted.

#### 2.3.2 Independent variables

The independent variables in this study are grouped into individual-level and community-level factors.

*Individual-level factors* include characteristics of both the mother and child, such as the child’s sex and age, the mother’s age, education level, marital status, work status, antenatal care, place of delivery, as well as the child’s birth order.

*Household and environmental factors*, such as household size, household wealth, type of toilet facility, and the source of drinking water, are also crucial for understanding the impact of these factors on ICU.

*Community-level factors* includes place of residence, the illiteracy level within the community, the community’s wealth, and the region. These factors help provide context for how the surrounding community environment might influence chronic malnutrition across generations in Ethiopia.

### 2.4 Spatial autocorrelation

Moran’s I was calculated using ArcGIS version 10.8 to assess the spatial autocorrelation of ICU. The Global Moran’s I index ranges from −1 to +1, where values close to −1 indicate a dispersed or scattered pattern of ICU prevalence, while values near +1 suggest a clustered pattern (32). A value of 0 represents a random distribution of ICU. To determine the statistical significance of the spatial autocorrelation, a *p*-value threshold of less than 0.05 was applied. This approach helped identify areas with significant spatial patterns in the prevalence of ICU.

### 2.5 Hot spot analysis

The spatial autocorrelation between regions was analyzed using hot spot analysis, specifically the Getis-Ord Gi* statistic, to identify areas with significant clusters of ICU in Ethiopia. This analysis helped highlight regions where both maternal and child stunting were more prevalent than expected, as well as areas with lower-than-expected rates of ICU. By examining these spatial patterns, the hot spot analysis provided valuable insights into which regions were most affected by ICU, enabling better-targeted interventions. The Getis-Ord Gi* statistic revealed whether areas with high or low rates of ICU tended to be grouped together, or if they were spread out randomly (33). This approach helped identify high-risk areas that require more focused attention and resources to combat malnutrition across generations.

### 2.6 Spatial interpolation

Due to limited resources and time, it was challenging to assess ICU prevalence among women with children aged 0–59 months across all areas of the country. As a result, predicting ICU in unsampled areas based on sampled data became essential. To address this, the ordinary kriging spatial interpolation technique was applied, under the assumption that nearby areas are more likely to exhibit similar patterns of ICU than distant ones (34). This approach allowed for a more comprehensive prediction of ICU prevalence across unsampled regions.

### 2.7 Spatial scan statistics

Spatial scan statistics were used to identify significant clusters of stunting among children aged 6–23 months in Ethiopia. The analysis utilized Kulldorff’s SaTScan version 10.1 software, applying Bernoulli-based methods with a default maximum spatial cluster size of 50% to scan the study area (35). This method systematically examines the geographic distribution of stunting by scanning with varying window sizes and shapes to detect regions with higher or lower prevalence compared to the national average. Statistical significance was determined using a likelihood ratio test with a *p*-value threshold of 0.05. The analysis is crucial for identifying both high-risk areas offering essential insights for targeting interventions where they are most needed.

### 2.8 Multilevel analysis

A multilevel binary logistic regression model was employed to identify potential risk factors for ICU among mothers with children aged 0–59 months in Ethiopia using STATA-17. The analysis accounted for the hierarchical structure of the data, where individuals and households were nested within enumeration areas (EAs) (36).

Consequently, a two-level model was adopted, with secondary sampling units (individuals and households) designated as level-one units and primary sampling units (EAs) as level-two units. The multilevel binary logistic regression model included fixed effects and cluster-specific random effects to address within-cluster correlation in the data. Four models were considered for the multilevel analysis:

Model I (empty model) was fitted without explanatory variables to test random variability.
Model II examined the effects of individual level characteristics.
Model III examined the effect of community level variables.
Model IV (full model) examined the effects of both individual and community level characteristics simultaneously. In the random-effects model, we computed intra-class correlation coefficient (ICC), median odds ratio (MOR), and proportional change in variance (PCV) statistics for measures of variation between clusters (37).

The ICC quantifies the variation of ICU among women with children aged 0–59 months within clusters in Ethiopia (38). The ICC may range from 0 to 1. Intra-class correlation coefficient = 0 showed perfect independence of residuals and the observations do not depend on clusters. However, ICC = 1 or less than one indicates interdependence of residuals, i.e., the variation of observations between clusters (37). It is calculated using the formula:


$$\mathrm{ICC}\;(\rho )=\frac{\sigma^{2}\varepsilon }{\sigma^{2}\mu +\sigma^{2}\varepsilon }$$

where: *σ*^2^*ε* is the within-group (or residual) variance and *σ*^2^*μ* is the between-group variance.

The median odds ratio (MOR) is defined as the median value of the odds ratio comparing the area with the highest risk to the area with the lowest risk when two individuals are selected from two different, randomly chosen clusters. It quantifies the unexplained cluster-level heterogeneity in the model. The MOR is calculated using the following formula (37):


$$\mathrm{MOR}=\exp[\sqrt{\left(2*\mathrm{VA})*0.6745\right]}=\exp(0.95\sqrt{\mathrm{VA}})$$

where; VA is the area level variance, and 0.6745 is the 75th centile of the cumulative distribution function of the normal distribution with mean 0 and variance 1. The MOR is always greater than or equal to 1. If the MOR is 1, there is no variation between clusters.

The total variation attributed to individual and cluster level factors at each model was measured by the PCV and it is calculated as the following formula (37):


PCV=(VA−VB)/VA∗100

where VA = variance of the initial model, and VB = variance of the model with more terms.

### 2.9 Ethical considerations

Approval for using the EDHS dataset was obtained from the DHS Program/ICF International Inc., and the IRB of the DHS Program https://www.dhsprogram.com. The dataset is de-identified with randomized geographic coordinates to ensure privacy. No additional ethical review was required as there was no direct interaction with participants. We followed all DHS Program ethical guidelines.

## 3 Results

### 3.1 Socio-demographic and economic characteristics of participants

From 2000 to 2016, a total of weighted of 33,445 mothers with children aged 0–59 months and their households were included in the analysis. Male children accounted for 51.10% of the sample. Most households (89.23%) were in rural areas, with 57.19% having five or fewer members. Families with two or more children under 5 years constituted 65.85% of the total. Regarding maternal characteristics, 50.80% of the mothers were aged 25–34 years, and 72.94% had no formal education.

Over half (51.43%) of the mothers were employed, and 91.57% of the mothers were married. Male-headed households made up 86.58% of the total, while 45.28% of households were categorized as low-income. Most births (87.44%) occurred at home, and unimproved toilet facilities were prevalent (88.99%). A significant portion of households (84.13%) had non-piped water sources, and 60.94% of mothers attended at least one antenatal visit. Children born with a birth order of 1–3 represented 48.58% of the sample (Table 1).

**Table 1:** Socio-demographic and household characteristics of mothers and children aged 0–59 months in Ethiopia, 2000–2016.

Characteristics	Category	2000	2005	2011	2016	2000–2016
		*n* (%)	*n* (%)	*n* (%)	*n* (%)	*n* (%)
Sex of child	Male	4,967 (50.72)	2,190 (50.89)	5,122 (51.33)	4,811 (51.35)	17,091 (51.10)
	Female	4,826 (49.28)	2,114 (49.11)	4,857 (48.67)	4,558 (48.65)	16,354 (48.90)
Place of residence	Urban	1,011 (10.33)	325 (7.56)	1,237 (12.40)	1,028 (10.97)	3,602 (10.77)
	Rural	8,782 (89.67)	3,978 (92.44)	8,741 (87.60)	8,342 (89.03)	29,843 (89.23)
Number of household members	More than 5 members	4,209 (42.98)	1,675 (38.92)	4,376 (43.85)	4,058 (43.31)	14,318 (42.81)
	5 or fewer members	5,584 (57.02)	2,629 (61.08)	5,603 (56.15)	5,311 (56.69)	19,127 (57.19)
Number of children under 5	2 or more children	6,518 (66.55)	2,978 (69.20)	6,625 (66.39)	5,902 (63.00)	22,023 (65.85)
	Less than 2 children	3,276 (33.45)	1,325 (30.80)	3,353 (33.61)	3,467 (37.00)	11,421 (34.15)
Mother’s age	15–24	2,544 (25.97)	1,030 (23.94)	2,351 (23.56)	2,063 (22.02)	7,988 (23.89)
	25–34	4,646 (47.44)	2,116 (49.16)	5,196 (52.07)	5,031 (53.70)	16,989 (50.80)
	35–49	2,603 (26.58)	1,157 (26.89)	2,432 (24.37)	2,275 (24.28)	8,467 (25.32)
Maternal education level	No education	7,989 (81.58)	3,358 (78.04)	6,903 (69.18)	6,143 (65.57)	24,394 (72.94)
	Primary education	1,291 (13.18)	750 (17.43)	2,715 (27.21)	2,571 (27.44)	7,326 (21.91)
	Secondary and above	513 (5.24)	195 (4.53)	361 (3.62)	655 (6.99)	1,724 (5.16)
Maternal work status	Unemployed	3,551 (36.26)	2,972 (69.07)	4,554 (45.64)	5,167 (55.15)	16,244 (48.57)
	Employed	6,242 (63.74)	1,331 (30.93)	5,424 (54.36)	4,202 (44.85)	17,200 (51.43)
Marital status	Married	9,024 (92.15)	4,008 (93.14)	8,788 (88.07)	8,804 (93.97)	30,625 (91.57)
	Unmarried	769 (7.85)	295 (6.86)	1,190 (11.93)	565 (6.03)	2,819 (8.43)
Sex of household head	Male	8,516 (86.96)	3,788 (88.02)	8,557 (85.76)	8,095 (86.39)	28,955 (86.58)
	Female	1,278 (13.04)	516 (11.98)	1,421 (14.24)	1,275 (13.61)	4,489 (13.42)
Wealth index	Low-income	0 (0)	1,896 (44.05)	4,458 (44.68)	4,355 (46.48)	10,708 (45.28)
	Middle-income	0 (0)	888 (20.63)	2,093 (20.97)	1,971 (21.03)	4,951 (20.93)
	High-income	0 (0)	1,520 (35.32)	3,428 (34.35)	3,044 (32.49)	7,992 (33.79)
Place of delivery	Home	9,296 (94.92)	4,086 (94.95)	9,012 (90.32)	6,851 (73.12)	29,245 (87.44)
	Health facility	498 (5.08)	217 (5.05)	966 (9.68)	2,518 (26.88)	4,199 (12.56)
Current age of child	0–11 months	1,929 (19.69)	834 (19.38)	2,047 (20.51)	2,005 (21.40)	6,814 (20.38)
	12–23 months	2,029 (20.71)	812 (18.87)	1,806 (18.10)	1,868 (19.94)	6,517 (19.49)
	24–35 months	1,676 (17.12)	728 (16.93)	1,519 (15.22)	1,527 (16.29)	6,458 (19.31)
	36–47 months	1,747 (17.84)	724 (16.82)	1,925 (19.30)	1,546 (16.50)	6,978 (20.86)
	48–59 months	2,413 (24.64)	1,205 (28.01)	2,681 (26.87)	2,424 (25.87)	6,678 (19.97)
Type of toilet facility	Improved	1,360 (13.89)	218 (5.06)	1,219 (12.22)	885 (9.44)	3,682 (11.01)
	Unimproved	8,433 (86.11)	4,085 (94.94)	8,759 (87.78)	8,484 (90.56)	29,762 (88.99)
Source of drinking water	Non-piped	8,545 (87.25)	3,608 (83.83)	7,418 (74.34)	8,567 (91.44)	28,138 (84.13)
	Piped	1,248 (12.75)	696 (16.17)	2,561 (25.66)	802 (8.56)	5,307 (15.87)
Number of antenatal visits	No	4,856 (49.58)	1,938 (45.03)	3,887 (38.96)	2,383 (25.43)	13,064 (39.06)
	Yes	4,938 (50.42)	2,366 (54.97)	6,091 (61.04)	6,987 (74.57)	20,381 (60.94)
Birth order	1–3 BORD	4,736 (48.36)	1,950 (45.31)	4,951 (49.62)	4,611 (49.21)	16,248 (48.58)
	>3 BORD	5,058 (51.64)	2,354 (54.69)	5,027 (50.38)	4,758 (50.79)	17,197 (51.42)

BORD, birth order; DDS; dietary diversity score.

### 3.2 The prevalence of intergenerational chronic malnutrition and its regional distribution

**Figure 2:**
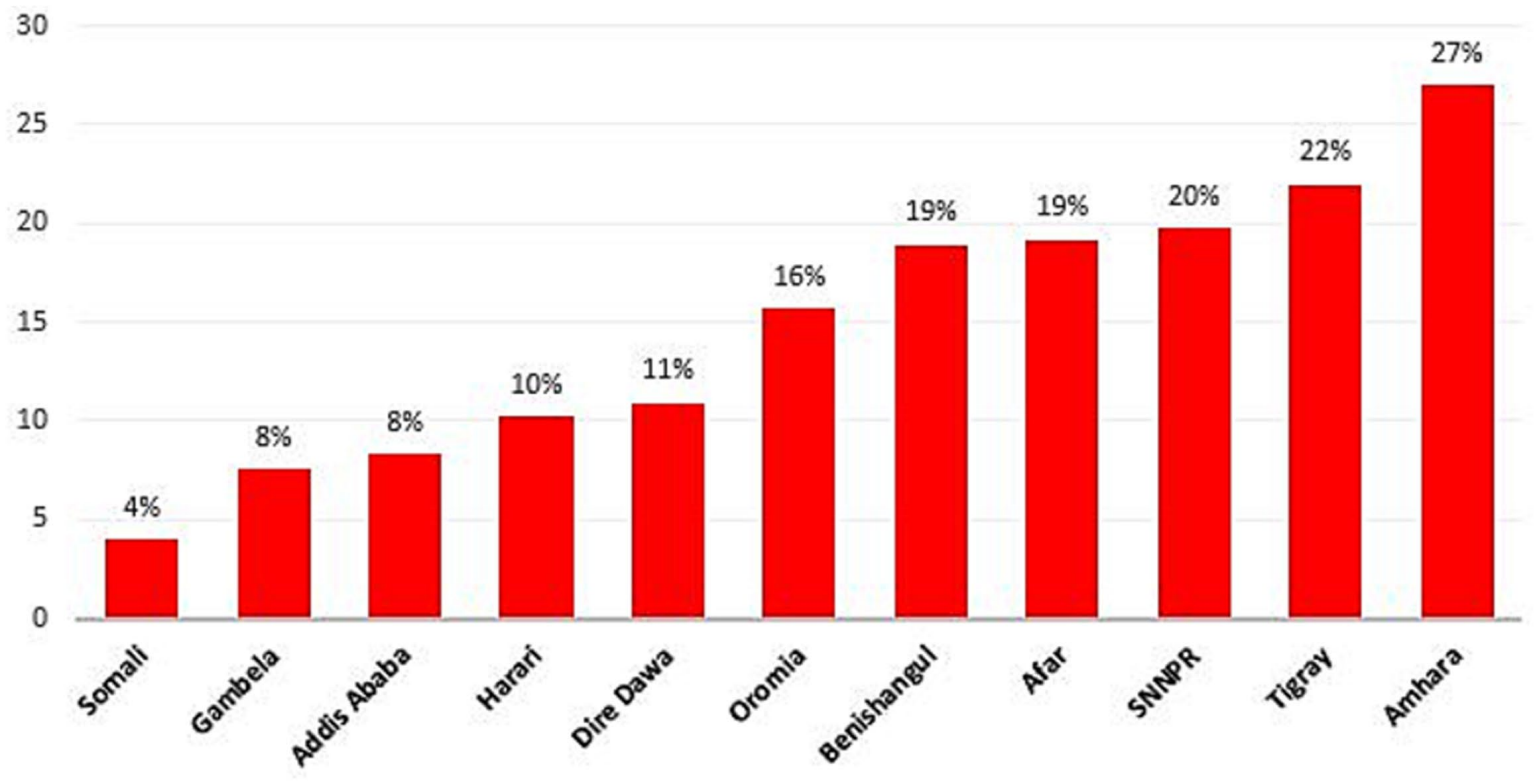
The prevalence of intergenerational chronic malnutrition across region in Ethiopia from 2000–2016.

The overall prevalence of intergenerational malnutrition (ICU) is 19.09% (95% CI: 18.68–19.52%). Among the regions, Amhara had the highest prevalence of ICU at 27%, followed by Tigray at 22% (Figure 2). In contrast, Somali had the lowest prevalence at 4%, with Gambela close behind at 7.5%. The prevalence of undernutrition (ICU) varied across both regions and years. In 2000, Amhara recorded the highest prevalence at 30.18%, while Somali had the lowest at 4.91%. By 2005, Amhara remained the highest at 28.99%, with Gambela showing the lowest at 6.59%. In 2011, Amhara still had the highest prevalence at 26.56%, while Gambela again had the lowest at 5.42%. By 2016, Amhara continued to show the highest prevalence at 21.80%, and Somali had the lowest at 1.87% (Table 2).

**Table 2:** Prevalence of intergenerational chronic undernutrition by region and year in Ethiopia: EDHS data from 2000 to 2016.

No.	Region	EDHS 2000		EDHS 2005		EDHS 2011		EDHS 2016	
		ICU prevalence %	Not ICU prevalence %	ICU prevalence %	Not ICU prevalence %	ICU prevalence %	Not ICU prevalence %	ICU prevalence %	Not ICU prevalence %
1.	Tigray	28.50	71.50	16.55	83.45	24.39	75.61	16.14	83.86
2.	Afar	21.86	78.14	15.86	84.14	19.38	80.62	21.22	78.78
3.	Amhara	30.18	69.82	28.99	71.01	26.56	73.44	21.80	78.20
4.	Oromia	18.28	81.72	17.23	82.77	14.92	85.08	12.53	87.47
5.	Somali	4.91	95.09	5.37	94.63	4.12	95.88	1.87	98.13
6.	Benishangul	17.72	82.28	19.31	80.69	20.29	79.71	18.27	81.73
7.	SNNPR	25.12	74.88	21.14	78.86	19.00	81.00	14.35	85.65
8.	Gambela	12.66	87.34	6.59	93.41	5.42	94.58	4.39	95.61
9.	Harari	11.44	88.56	13.15	86.85	8.84	91.16	9.46	90.54
10.	Addis Ababa	12.17	87.83	9.03	90.97	9.63	90.37	3.80	96.20
11.	Dire Dawa	11.16	88.84	8.93	91.07	12.20	87.80	10.29	89.71

### 3.3 Spatial analysis

#### 3.3.1 Spatial global autocorrelation

**Figure 3:**
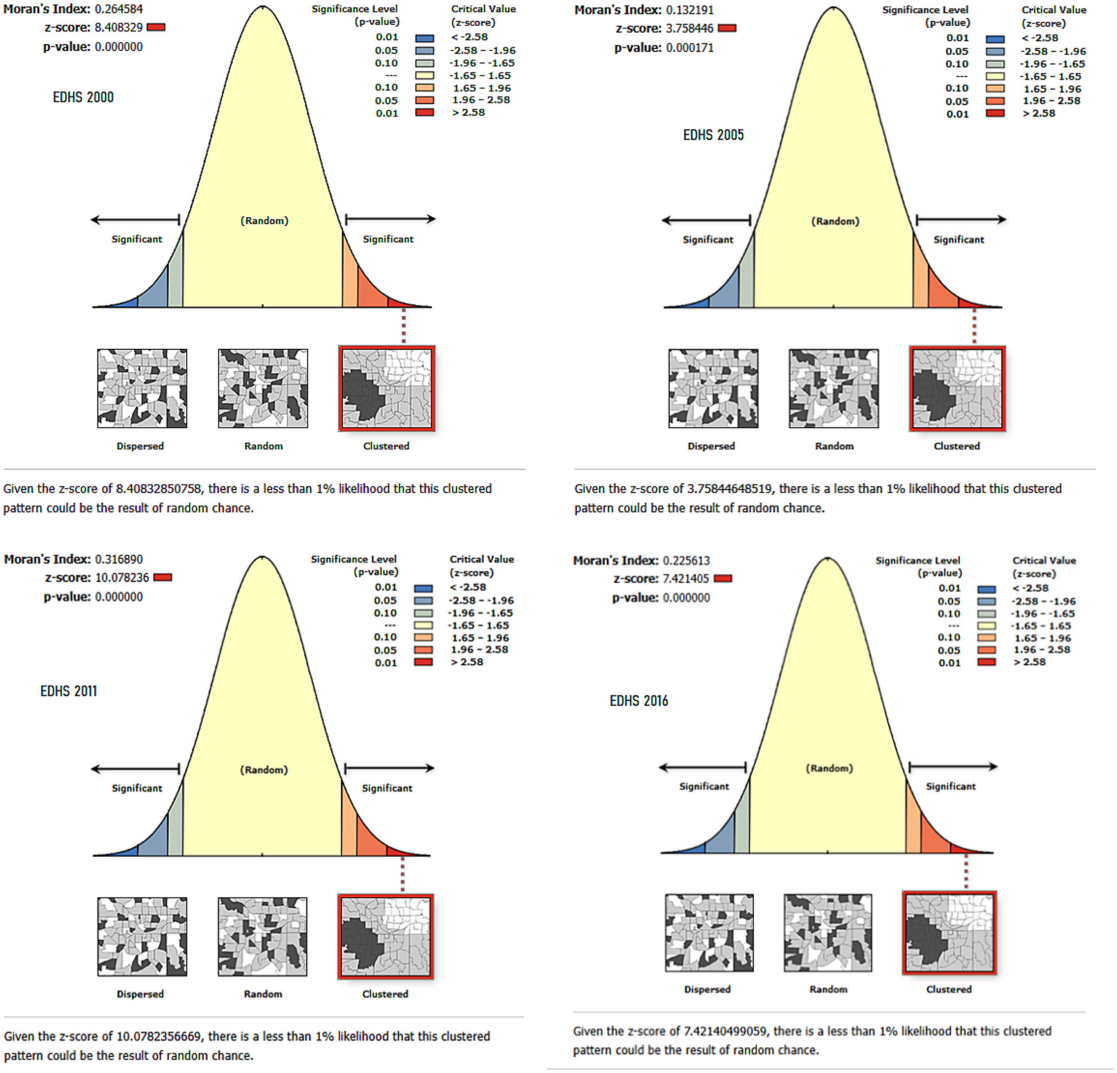
Spatial autocorrelation of intergenerational chronic undernutrition in Ethiopia: Moran’s Index Analysis (EDHS 2000–2016).

The analysis of ICU among women with children aged 0–59 months in Ethiopia, using EDHS data from 2000 to 2016, shows a persistent pattern of spatial clustering. Moran’s Index values were 0.246584 in 2000, 0.316890 in 2011, 0.132191 in 2005, and 0.225613 in 2016, all with highly significant *p*-values (*p* < 0.001). These findings confirm that chronic malnutrition is not randomly distributed but concentrated in specific regions. These results highlight the need for targeted regional interventions to address the burden of chronic malnutrition in Ethiopia (Figure 3).

#### 3.3.2 Hotspot analysis of intergenerational chronic malnutrition in Ethiopia

**Figure 4:**
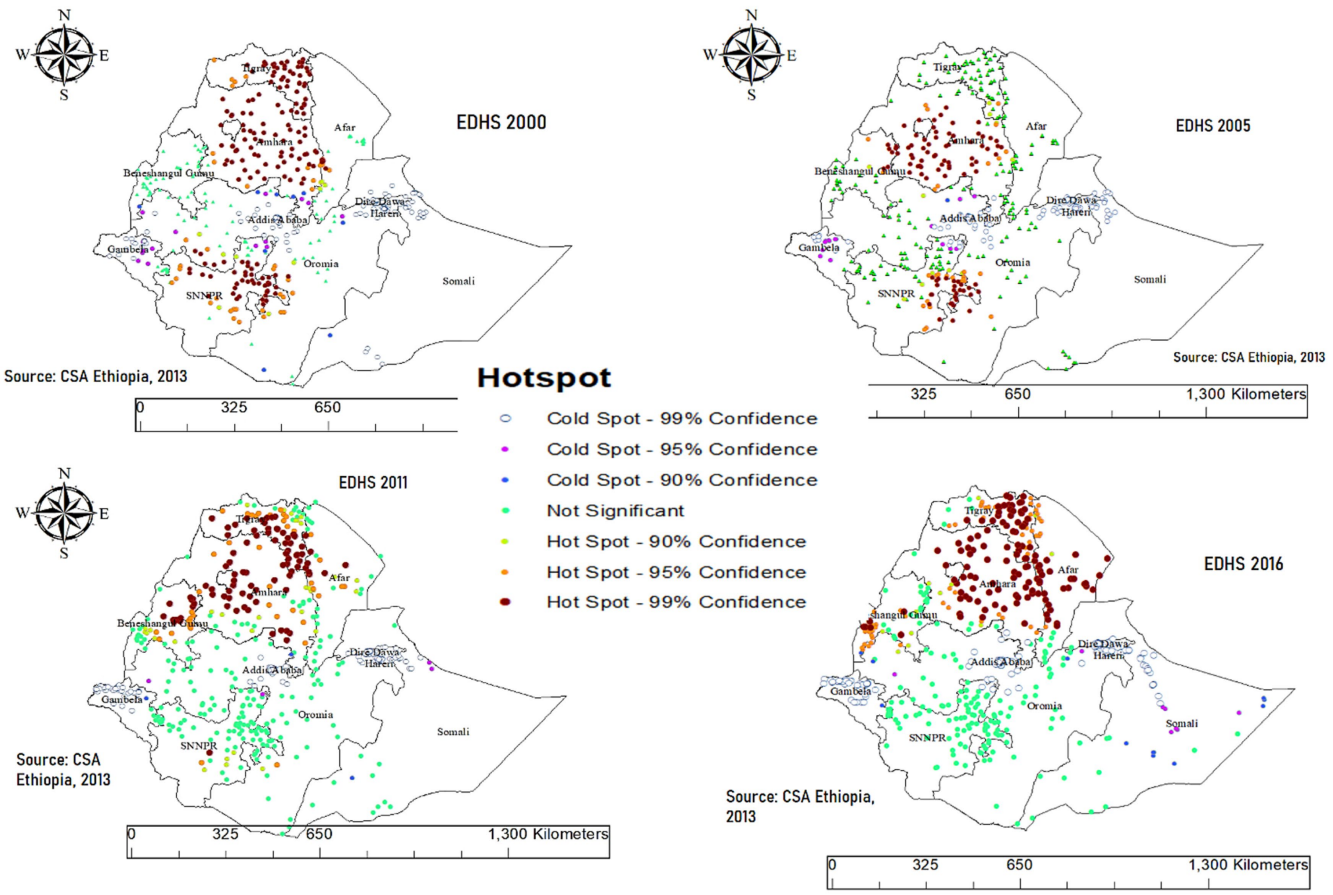
Hotspot and cold spot analysis of chronic malnutrition among children aged 0–59 months in Ethiopia (EDHS 2000–2016).

The hotspot areas for ICU are predominantly located in the northern and northeastern regions of Ethiopia, including Tigray, Amhara, and parts of Afar. These regions consistently show clustering of malnutrition across all survey years. To a lesser extent, SNPPR, parts of Oromia and Benishangul-Gumuz also exhibited hotspot areas, particularly in 2000 and 2011 surveys. Conversely, cold spot areas are mainly observed in the southeastern and southern regions, such as Somali, southern parts of the SNNPR, and occasionally in Gambela. In 2005, cold spot areas were predominantly observed across significant parts of Tigray, Afar, as well as portions of Oromia and SNNPR. The Harari and Dire Dawa areas also showed limited cold spots (Figure 4). These patterns highlight a persistent geographic disparity in malnutrition, with northern regions being more affected compared to the southern and southeastern parts of the country.

#### 3.3.3 Spatial interpolation of intergenerational chronic malnutrition in Ethiopia

**Figure 5:**
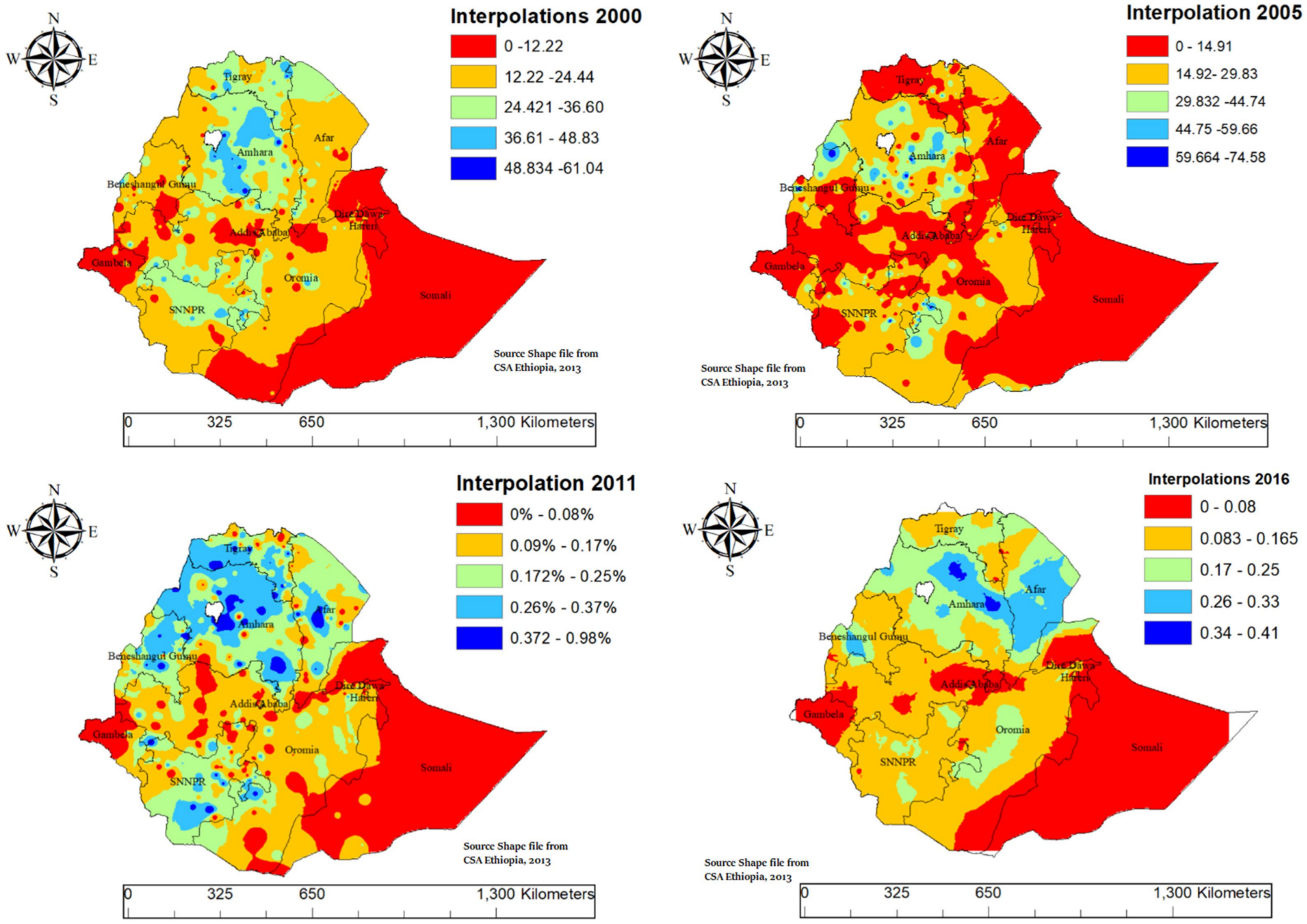
Spatio-temporal interpolation of intergenerational chronic undernutrition in Ethiopia (2000–2016).

Ordinary Kriging interpolation of ICU from unsampled areas in the EDHS showed the highest predictions in the Somali region and southeastern parts of Oromia across all time points (2000, 2005, 2011, and 2016). These areas, highlighted in red and orange, consistently represent the highest predicted risk of ICU. Additional high-prediction areas were observed in parts of SNNPR, including its southwestern zones. In contrast, the lowest predicted risks, shown in blue, were found in Addis Ababa, its surrounding areas in Oromia, and some parts of northern Ethiopia, including portions of Tigray and Amhara. These regions consistently exhibited lower ICU predictions throughout the years, emphasizing the spatial disparities in chronic malnutrition across the country (Figure 5).

#### 3.3.4 Spatial scan statistical analysis of intergenerational chronic malnutrition

In spatial scan statistical analysis in EDHS 2000 the primary significant big cluster of spatial windows encompassed most parts of Amhara, Tigray, most parts of Afar, and Northern part of Somali. The cluster was identified at coordinates (11.887378°N, 39.448211°E) with a radius of 242.76 km. The relative risk, log likelihood ratio, and *p*-value were (RR = 1.58, LLR = 69.77, *p*-value = 0.001), indicating a significantly higher risk of ICU in this cluster compared to areas outside. In EDHS 2005 the primary significant big cluster of spatial windows encompassed most parts of Amhara. The primary cluster was identified at coordinates (11.529743°N, 38.342559°E) with a radius of 185.26 km. The relative risk, log likelihood ratio, and *p*-value were (RR = 1.86, LLR = 43.36, *p*-value = 0.001), indicating a significantly higher risk of the target variable in this cluster compared to areas outside.

**Figure 6:**
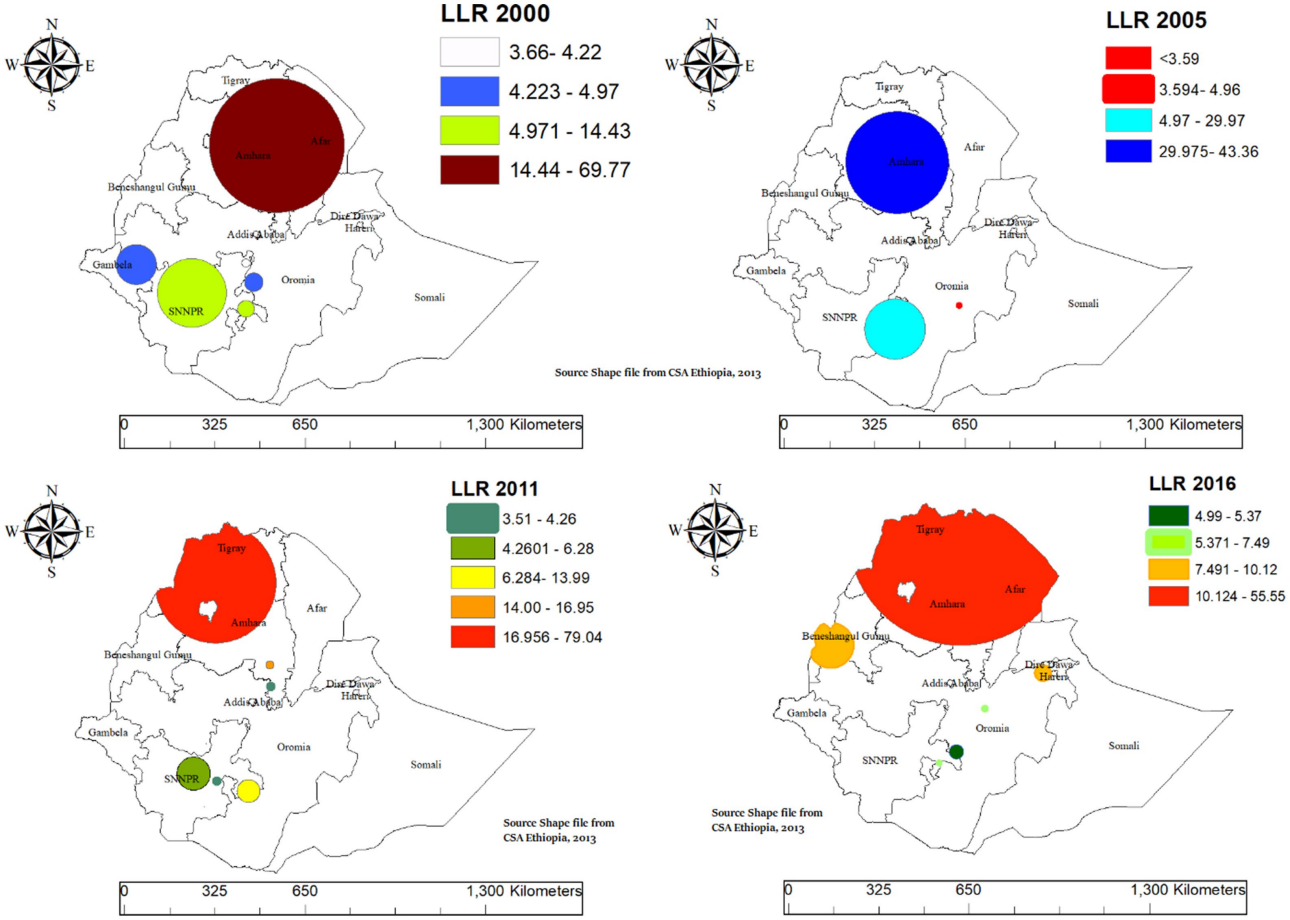
SaTScan cluster analysis of intergenerational chronic undernutrition among women with aged 0–59 months late-adolescent girls in Ethiopia, 2000–2016.

The primary cluster in the EDHS 2011 dataset was identified at coordinates (12.859670°N, 37.548771°E) with a radius of 217.83 km, encompassing most parts of Amhara, Tigray, and northern Benishangul. The relative risk, log likelihood ratio, and *p*-value were (RR = 1.83, LLR = 79.04, *p*-value = 0.001), indicating a significantly higher risk of the target variable in this cluster compared to areas outside. The primary cluster in the EDHS 2016 dataset was identified at coordinates (13.834595°N, 39.094466°E) with a radius of 402.27 km. This cluster encompasses all parts of Tigray, most parts of Amhara, and all parts of Afar. The relative risk, log likelihood ratio, and *p*-value were (RR = 1.75, LLR = 55.55, *p*-value = 0.001), indicating a significantly higher risk of the target variable in this cluster compared to areas outside (Table 3 and Figure 6).

**Table 3:** Significant clusters of SaTScan analysis for intergenerational chronic malnutrition among women with children aged 0–59 months in Ethiopia, EDHS 2000 to 2016.

	Cluster type	Location IDs included	Coordinates/Radius (km)	Population	Cases	RR	LLR	*p*-value
EDHS 2000	Primary	107, 103, 108, 104, 111, 102, 106, 155, 41, 43, 105, 42, 112, 156, 39, 146, 110, 151, 109, 159, 114, 40, 48, 38, 116, 97, 115, 82, 145, 36, 113, 93, 67, 68, 98, 66, 150, 123, 73, 119, 70, 69, 72, 71, 117, 37, 99, 96, 74, 130, 129, 95, 35, 33, 118, 100, 122, 128, 58, 24, 59, 60, 50, 75, 92, 34, 49, 61, 120, 152, 101, 124, 154, 23, 84, 94, 85, 76, 21, 77, 81, 131, 88, 30, 6, 22, 87, 136, 29, 32, 78, 125, 55, 80, 54, 142, 52, 11, 47, 158, 51, 56, 57, 20, 127, 53, 79, 139, 138, 132, 140, 194, 31, 19, 135, 153, 12, 64, 28, 121, 89, 196, 195, 25	(11.887378°N, 39.448211°E)/242.76 km	2,713	854	1.58	69.77	0.001
	Secondary	343, 342, 344, 341, 340, 345, 348	(6.563394°N, 38.436912°E)/30.10 km	243	93	1.69	14.43	0.001
	Tertiary	351, 373, 181, 180, 182, 359, 374, 372, 360, 358, 179, 177, 357, 362, 368, 361, 178, 183, 371, 370, 353, 377, 236, 384, 365, 352, 334, 326, 364, 363	9.001887°N, 38.709463°E/8.48	970	281	1.28	9.66	0.039
EDHS 2005	Primary	27, 350, 479, 351, 326, 152, 418, 483, 192, 461, 338, 149, 262, 304, 354, 110, 99, 115, 288, 102, 182, 307, 17, 74, 231, 24, 145, 22, 159, 97, 225, 415, 96, 264, 66, 277, 181, 396, 1, 349, 187, 128, 190, 278, 402, 364, 322, 393, 427, 382, 428, 528, 447, 156, 125, 516, 29, 75, 211, 463, 244, 216, 71, 101, 511, 423, 330, 15, 204, 214, 98, 76, 103, 147, 458, 414, 250	(11.529743°N, 38.342559°E)/185.26 km	908	286	1.86	43.36	0.001
	Secondary	444, 234, 189, 61, 77, 280, 40, 90, 258, 146, 206, 80, 172, 213, 317, 455, 68, 13, 489, 30, 7, 249, 343, 229, 12, 28, 161, 539, 252, 126, 53, 46, 353	(6.099264°N, 38.281627°E)/109.19 km	482	165	1.87	29.97	0.001
EDHS 2011	Primary	333, 154, 460, 316, 174, 122, 637, 334, 227, 271, 504, 592, 621, 347, 64, 462, 218, 123, 77, 403, 288, 274, 195, 245, 181, 188, 213, 249, 115, 217, 180, 35, 139, 620, 473, 183, 453, 369, 20, 634, 615, 86, 638, 551, 311, 538, 650, 280, 417, 39, 488, 148, 510, 556, 365, 226, 71, 597, 582, 46, 635, 89, 322, 426, 247, 241, 318, 225, 484, 406, 557, 224	(12.859670°N, 37.548771°E)/217.83 km	1,686	516	1.83	79.04	0.001
	Secondary	1, 219	(10.193657°N, 39.345773°E)/14.33 km	34	22	3.41	16.94	0.001
	Tertiary	518, 320	(6.084820°N, 38.650426°E)/41.31 km	117	47	2.13	13.98	0.002
	Quaternary	284	(5.570489°N, 37.212779°E)/0 km	38	21	2.91	12.28	0.005
EDHS 2016	Primary	355, 604, 481, 430, 579, 461, 226, 575, 129, 45, 84, 156, 636, 81, 341, 590, 94, 237, 550, 220, 538, 598, 623, 400, 424, 404, 605, 597, 551, 99, 298, 89, 196, 384, 479, 117, 160, 192, 584, 413, 127, 263, 421, 362, 181, 103, 340, 425, 235, 188, 134, 585, 98, 255, 392, 528, 511, 79, 542, 143, 172, 130, 449, 78, 136, 128, 80, 583, 300, 628, 258, 442, 268, 322, 97, 351, 66, 488, 199, 152, 249, 312, 455, 612, 200, 327, 599, 638, 544, 401, 344, 332, 640, 253, 296, 478, 241, 591, 504, 163, 389, 348, 189, 496, 627, 132, 571, 512, 427, 191, 410, 545, 158, 279, 611, 292, 38, 354, 345, 456, 254, 616, 169, 18, 368, 73, 205, 178, 499, 617, 570, 431, 167, 55, 176, 334, 403, 120, 24, 516, 382, 460, 429, 547, 440, 206, 632, 52, 10, 596, 366, 276, 361, 267, 4, 259, 620, 482, 602, 75, 109, 310, 3, 229	(13.834595°N, 39.094466°E)/402.27 km	2,318	504	1.75	55.55	0.001
	Secondary	453, 557, 441, 594, 166, 30, 473	(9.303716°N, 41.792392°E)/29.03 km	152	44	1.99	10.12	0.021
	Tertiary	209, 563, 407, 595, 409, 433, 569, 335, 203, 6, 581, 317, 416, 508, 165, 65, 17, 457, 285, 374, 124, 462, 621	(10.189214°N, 34.841634°E)/81.37 km	123	37	2.06	9.41	0.037

RR, relative risk; LLR, log likelihood ratio.

### 3.4 Multilevel analysis

#### 3.4.1 Individual and community level factors

We conducted a multilevel analysis to identify the factors contributing to ICU among women with children aged 0–59 months, incorporating both individual and community-level variables. The selection of variables was based on a bivariable analysis with a significance threshold of 0.2. The analysis was carried out in four stages:

Model I (Empty Model) assessed the variance across communities.
Model II included only individual-level factors.
Model III focused on community-level factors.
Model IV combined both individual and community-level factors. This stepwise approach provided a comprehensive understanding of the multilevel factors influencing ICU.

*Based on the individual-level model (model II)*, several factors were significantly associated with ICU. Children from the richest households Protestant (AOR = 0.64; 95% CI: 0.57–0.73) and Muslim households (AOR = 0.66; 95% CI: 0.59–0.73), secondary or higher education (AOR = 0.35; 95% CI: 0.27–0.45), women with children from primary education (AOR = 0.88; 95% CI: 0.79–0.98), high-income households (AOR = 0.83; 95% CI: 0.75–0.93), children aged 12–23 months (AOR = 3.93; 95% CI: 3.37–4.58), 24–35 months (AOR = 3.36; 95% CI: 2.88–3.92), 36–47 months (AOR = 3.68; 95% CI: 3.16–4.28), and 48–59 months (AOR = 5.55; 95% CI: 3.06–4.14) and households with unimproved toilet (AOR = 1.54; 95% CI: 1.33–1.79) were significantly associated with ICU.

*Based on the community-level model (model III)*, children from rural areas had significantly higher odds of ICU (AOR = 1.68; 95% CI: 1.48–1.34) compared to those from urban areas. Regional variations were also observed. Children from Amhara (AOR = 1.27; 95% CI: 1.12–1.43) had higher odds of ICU, while children from Oromia (AOR = 0.64; 95% CI: 0.57–0.72), Benshangul-Gumuz (AOR = 0.79; 95% CI: 0.68–0.91), SNPPR (AOR = 0.84; 95% CI: 0.75–0.95), Gambela (AOR = 0.23; 95% CI: 0.17–0.31), Harari (AOR = 0.44; 95% CI: 0.36–0.54), Addis Ababa (AOR = 0.52; 95% CI: 0.41–0.62), Somali (AOR = 0.12; 95% CI: 0.08–0.15) and Dire Dawa (AOR = 0.52; 95% CI: 0.43–0.62) had lower odds compared to Tigray.

*In model IV*, several factors were significantly associated with ICU. Female children had 7% lower odds of experiencing ICU compared to male children (AOR = 0.93; 95% CI: 0.86–0.99). Children whose mothers had secondary or higher education were 58% less likely to experience ICU compared to those whose mothers had no formal education (AOR = 0.42; 95% CI: 0.32–0.54). The wealth index was a significant factor in ICU, with children from middle-income households being 14% less likely to experience ICU (AOR = 0.86; 95% CI: 0.76–0.96), and children from high-income households being 16% less likely to experience ICU compared to those from low-income households (AOR = 0.84; 95% CI: 0.75–0.94).

The child’s age was a strong factor of ICU, with older children being much more likely to experience it compared to those aged 0–11 months. Children aged 12–23 months were almost four times more likely to be affected (AOR = 3.93; 95% CI: 3.37–4.58). Similarly, children aged 24–35 months had more than three times the risk (AOR = 3.42; 95% CI: 2.94–4.00), those aged 36–47 months were 3.67 times more likely to experience ICU (AOR = 3.67; 95% CI: 3.15–4.27), and children aged 48–59 months had 3.62 times the odds of ICU (AOR = 3.62; 95% CI: 3.11–4.23) compared to the youngest group.

The type of toilet facility was also an important factor in ICU. Children from households with unimproved toilet facilities were 1.26 times more likely to experience ICU compared to those with improved facilities (AOR = 1.26; 95% CI: 1.08–1.46). Regional differences played a significant role in ICU. Children from Amhara were 25% more likely to experience ICU compared to those in Tigray (AOR = 1.25; 95% CI: 1.06–1.46). In contrast, children from Somali (AOR = 0.12; 95% CI: 0.08–0.15), Oromia (AOR = 0.63; 95% CI: 0.51–0.76), Harari (AOR = 0.48; 95% CI: 0.36–0.63), Gambela (AOR = 0.23; 95% CI: 0.17–0.31), Addis Ababa (AOR = 0.59; 95% CI: 0.42–0.83), and Dire Dawa (AOR = 0.52; 95% CI: 0.39–0.68) were significantly less likely to experience ICU compared to those in Tigray (Table 4).

**Table 4:** Individual and community level factors of Intergenerational chronic malnutrition in Ethiopia 2000–2016.

Characteristics	Category	ICU		AOR (95% CI)			
		No	Yes	Model I (empty)	Model II	Model III	Model IV
Individual level factors							
Sex of child	Male	13,698	3,392		1		1
	Female	13,360	2,994		0.92 (0.85, 1.01)		0.93 (0.86, 0.99)^*^
Number of household members	More than 5 members	15,464	3,663		1		1
	5 or fewer members	11,594	2,724		0.95 (0.86, 1.05)		0.98 (0.89, 1.09)
Religion	Orthodox	10,692	3,044		1		1
	Catholic	238	64		0.86 (0.57, 1.32)		1.21 (0.79, 1.88)
	Protestant	5,681	1,223		0.64 (0.57, 0.73)^**^		0.89 (0.76, 1.06)
	Muslim	9,723	1,924		0.66 (0.59, 0.73)^**^		1.06 (0.93, 1.21)
	Others	724	133		0.62 (0.46, 0.84)^**^		0.83 (0.60, 1.14)
Mother’s age	15–24	6,513	1,475		1		1
	25–34	13,732	3,257		0.96 (0.86, 1.06)		0.97 (0.87, 1.08)
	35–49	6,813	1,655		0.89 (0.77, 1.03)		0.87 (0.77, 1.03)
Maternal education level	No education	19,301	5,093		1		1
	Primary education	6,158	1,169		0.88 (0.79, 0.98)^*^		0.93 (0.82, 1.01)
	Secondary and above	1,599	125		0.35 (0.27, 0.45)^**^		0.42 (0.32, 0.54)^**^
Maternal work status	Unemployed	13,392	2,853		1		1
	Employed	13,666	3,533		1.07 (0.99, 1.16)		0.98 (0.89, 1.07)
Marital status	Married	24,815	5,810		1		1
	Unmarried	2,243	576		1.06 (0.92, 1.22)		1.11 (0.97, 1.28)
Wealth index	Low-income	8,581	2,127		1		1
	Middle-income	4,120	831		0.93 (0.83, 1.04)		0.86 (0.76, 0.96)^**^
	High-income	6,837	1,154		0.83 (0.75, 0.93)^**^		0.84 (0.75, 0.94)^**^
Place of delivery	Home	23,412	5,833		1		1
	Health facility	3,646	553		0.83 (0.73, 0.95)^**^		0.92 (0.79, 1.04)
Current age of child	0–11 months	6,307	508		1		1
	12–23 months	5,060	1,457		3.93 (3.37, 4.58)^**^		3.93 (3.37, 4.58)^**^
	24–35 months	5,109	1,349		3.36 (2.88, 3.92)^**^		3.42 (2.94, 4.00)^**^
	36–47 months	5,385	1,592		3.68 (3.16, 4.28)^**^		3.67 (3.15, 4.27)^**^
	48–59 months	5,197	1,480		5.55 (3.06, 4.14)^**^		3.62 (3.11, 4.23)^**^
Type of toilet facility	Improved	3,114	568		1		1
	Unimproved	23,944	5,818		1.54 (1.33, 1.79)^*^		1.26 (1.08, 1.46)^**^
Source of drinking water	Non-piped	19,506	4,946		1		1
	Piped	7,552	1,441		0.93 (0.85, 1.02)		0.91 (0.82, 100)
Media exposure	No	1,713	4,420		1		1
	Yes	9,945	1,666		1.06 (0.97, 1.16)		1.02 (0.94, 1.12)
Birth order	1–3 BORD	13,265	2,983		1		1
	>3 BORD	13,793	3,404		1.06 (0.95, 1.18)		1.05 (0.94, 1.18)
Community level factors							
Place of residence	Urban	3,107	494			1	1
	Rural	23,950	5,892			1.68 (1.48, 190)^**^	1.11 (0.92, 134)
Illiteracy level	Low illiteracy	13,863	3,076			1	1
	High illiteracy	13,195	3,311			1.01 (0.93, 1.09)	0.99 (0.89, 1.10)
Community wealth level	Low wealth	11,807	2,835			1	1
	High wealth	15,251	3,552			1.00 (0.92, 1.09)	1.06 (0.95, 1.18)
Region	Tigray	1,780	508			1	1
	Afar	261	62			0.88 (0.77, 1.02)	0.83 (0.66, 1.03)
	Amhara	5,477	2,026			1.27 (1.12, 1.43)^**^	1.25 (1.06, 1.46)^**^
	Oromia	11,905	2,216			0.64 (0.57, 0.72)^**^	0.63 (0.51, 0.76)^**^
	Somali	876	36			0.12 (0.97, 0.16)^**^	0.12 (0.08, 0.15)^**^
	Benshangul-Gumuz	286	67			0.79 (0.68, 0.91)^**^	0.89 (0.73, 1.09)
	SNPPR	5,680	1,396			0.84 (0.75, 0.95)^**^	0.88 (0.72, 1.07)
	Gambela	79	7			0.27 (0.22, 0.33)^**^	0.23 (0.17, 0.31)^**^
	Harari	63	7			0.44 (0.36, 0.54)^*^	0.48 (0.36, 0.63)^**^
	Addis Ababa	547	48			0.52 (0.41, 0.62)^**^	0.59 (0.42, 0.83)^**^
	Dire Dawa	103	13			0.52 (0.43, 0.62)^**^	0.52 (0.39, 0.68)^**^

Measure of variation	Model I	Model II	Model III	Model IV
ICC %	8.72	6.48	2.60	4.25
PCV %	Reference	27.39	72.06	53.55
MOR	1.71	1.58	1.32	1.44
AIC	31917.61	17797.33	26357.4	17312.01
Log likelihood	−15956.80	−8872.665	−13163.7	−8617.00
BIC	31934.27	18005.41	26482.36	17624.13

*p<0.05, **p<0.01.

#### 3.4.2 Model fit and measures of variation

The intraclass correlation coefficient (ICC) decreased from 8.72% in Model I (the empty model) to 4.25% in Model IV, indicating a reduction in the variation explained by community-level factors. The PCV increased significantly from 27.39 to 53.55% in Model IV. The MOR decreased from 1.71 in Model I to 1.44 in Model IV, reflecting the reduction in community-level variation after adjusting for both individual and community-level factors. The Akaike information criterion (AIC) and Bayesian information criterion (BIC) indicated that Model IV, which included both individual and community-level factors, provided the best fit (AIC = 17312.01; BIC = 17624.13) (Table 4).

## 4 Discussion

This study aimed to investigate the prevalence, spatial distribution, and determinants of ICU in Ethiopia using spatial and multilevel analyses of EDHS data from 2000 to 2016. The findings revealed that the prevalence of ICU in Ethiopia was 19.09%, with the highest prevalence observed in the Amhara region and the lowest in the Somali region. Spatial analysis identified clusters of ICU, with the Amhara region consistently emerging as a persistent hotspot across all four surveys. Multilevel analysis highlighted various individual- and community-level factors associated with ICU.

The prevalence of ICU in Ethiopia is 19.09%, with regional variations: the highest in Amhara (27%), followed by Tigray (22%), and the lowest in Somali (4%). This highlights a significant public health concern driven by complex, multifactorial causes. Hotspot areas for ICU are concentrated in northern and northeastern regions, including Amhara, Tigray, and parts of Afar, consistently showing clustering across all survey years. Previous studies have consistently shown that these regions exhibit high rates of malnutrition among both children and women (39–41). This is likely due to a combination of factors, including widespread food insecurity, which limits access to adequate nutrition; poor maternal education, which affects knowledge and practices related to nutrition and child care; and recurrent droughts, which exacerbate food shortages and disrupt livelihoods, further contributing to the cycle of malnutrition in these regions (39, 42). Another possible explanation is that animal source food consumption is particularly low in the northern regions of Ethiopia, especially in Amhara and Tigray (43). This dietary inadequacy significantly contributes to undernutrition, as animal source foods are critical for providing essential nutrients like protein, iron, and vitamin B12, which are vital for growth and development (44, 45).

This study revealed that female children had 7% lower odds of experiencing ICU compared to male children. This finding consistent with previous studies conducted in different countries (46–49). Several explanations have been suggested for the observed gender differences in nutritional status. One possible reason is that boys tend to be more susceptible to infectious diseases and exhibit greater biological fragility, particularly during their first year of life (48, 50). This increased vulnerability may contribute to disparities in nutritional outcomes. Additionally, hormonal systems differ between boys and girls, and the interactions between sex hormones and environmental factors can influence energy consumption, nutritional requirements, and susceptibility to both infectious and noncommunicable diseases (51). While these factors may contribute to disparities in nutritional outcomes, the exact mechanisms underlying these biological differences remain largely unclear and warrant further investigation (52).

Children whose mothers attained secondary or higher education were 58% less likely to experience ICU compared to those whose mothers had no formal education. This result aligns with findings from previous studies which showed that maternal education has highly associated with undernutrition (53–57). This association may be explained by the fact that educated mothers often possess better knowledge of child nutrition, healthcare practices, and disease prevention. They are also more likely to access healthcare resources and exercise greater decision-making autonomy, leading to improved care and nutrition for their children (53, 55).

The wealth index was a significant factor in ICU, with children from middle-income households being 14% less likely to experience ICU. This finding is consistent with the results of prior studies conducted in various countries (58–61). Wealth status affects undernutrition as wealthier households can afford nutritious food, access better healthcare, and live-in healthier conditions, reducing the risk of malnutrition. In contrast, poorer families often face food insecurity, limited healthcare, and poor living conditions, which contribute to undernutrition (58, 59).

The child’s age was a strong factor of ICU, with older children being much more likely to experience it compared to those aged 0–11 months. The finding is consistent with different studies conducted previously which showed that older age children are more vulnerable for undernutrition (46, 62, 63). This finding suggests that older children are more vulnerable to ICU as their nutritional needs become more complex and they face greater exposure to environmental risks like poor sanitation and inadequate nutrition especially in low-income settings, may also contribute (63, 64). In addition, challenges such as food shortages from poor harvests or economic difficulties made older children more vulnerable to ICU. Studies showed low production of foods and financial hardship often leading to reduced food variety and availability in households, which can especially affect older children who need more diverse and nutritious diets to support their growth (65, 66).

Children from households with unimproved toilet facilities were 1.26 times more likely to experience ICU compared to those with improved facilities. This finding is in lined with previous studies conducted in Vietnam, Indonesia and Ethiopia (67–69). Poor sanitation, including unimproved toilet facilities, increases the risk of infections and diseases, which can contribute to undernutrition. These conditions often lead to waterborne diseases and poor hygiene, compromising children’s health. Improved latrines, typically associated with higher wealth, help prevent these risks by ensuring better sanitation and access to clean water, thus supporting better nutrition and health outcomes for children (69–71).

This study revealed that regional difference were a significant determinant of ICU among women with children aged 0–59 months. Children residing in Amhara had a 25% higher likelihood of experiencing ICU compared to those in Tigray. This finding is consistent with the results obtained in Ethiopia (41, 72, 73). The higher likelihood of ICU among children residing in Amhara compared to those in Tigray may be attributed to regional disparities in socioeconomic status, food security status. The Amhara region has consistently reported the highest percentage of food-insecure households in Ethiopia (74). Children from Somali, Oromia, Harari, Gambela, Addis Ababa, and Dire Dawa were significantly less likely to experience ICU than those from Tigray. This finding is supported by previous studies conducted in different parts of Ethiopia (75–77). Children in Tigray are more likely to experience ICU compared to those in other regions like Somali, Oromia, and Addis Ababa, mainly due to the area’s vulnerability to drought and limited agricultural production. Frequent droughts and environmental challenges have a severe impact on food security, as Tigray depends heavily on rain-fed agriculture (76, 78, 79). This makes the region especially sensitive to changing rainfall patterns, leading to lower crop yields and less access to nutritious food.

### 4.1 Strengths and limitations of this study

This study has several strengths, including using nationally representative EDHS data from 2000 to 2016, which helps capture trends over time, and applying both multilevel and spatial analyses to explore the factors influencing intergenerational chronic undernutrition (ICU) across regions. However, there are limitations as well. The study primarily focuses on maternal factors, leaving out potential paternal contributions. Because the data is cross-sectional, we cannot make definitive causal relationship. Additionally, the EDHS data did not collect all potential nutritional or environmental exposures, so the study could not explore these factors that may also play a role in ICU.

## 5 Conclusion and recommendations

The findings from this analysis highlight key factors influencing ICU in Ethiopia. The overall prevalence of ICU is notably high, with significant regional hotspots in the northern and northeastern parts of the country, particularly in Tigray, Amhara, and parts of Afar. The spatial scan analysis further identifies these regions as areas with concentrated risk. Factors such as female gender, maternal education (secondary or higher), and higher household wealth are associated with a reduced likelihood of experiencing ICU. In contrast, older child age, unimproved toilet facilities, and residing in Amhara were found to increase the likelihood of ICU. Regional variations also play a critical role, with children from Somali, Oromia, Harari, Gambela, Addis Ababa, and Dire Dawa showing a lower risk of ICU compared to those from Tigray. These findings suggest that improving education, especially maternal education, and expanding access to sanitation are essential to reduce ICU in Ethiopia. Addressing regional disparities through targeted interventions will help ensure more equitable health and nutrition outcomes, further reducing ICU.

## Data Availability

The datasets presented in this study can be found in online repositories. The names of the repository/repositories and accession number(s) can be found below: https://dhsprogram.com/Data/.
